# Where Dinner Roams: The Role of Feral Horses as a Resource Subsidy for Wolves and Cougars in West‐Central British Columbia

**DOI:** 10.1002/ece3.73089

**Published:** 2026-02-10

**Authors:** Shane C. White, Julie Thomas, Caroyln Shores, Kathi Zimmerman

**Affiliations:** ^1^ British Columbia Ministry of Water Land and Resource Stewardship Williams Lake British Columbia Canada; ^2^ Department of Ecosystem Science and Management University of Northern British Columbia Prince George British Columbia Canada; ^3^ British Columbia Ministry of Water Land and Resource Stewardship British Columbia Canada

**Keywords:** apparent competition, large carnivores, non‐native prey, predator–prey relationships

## Abstract

Feral horses ( *Equus ferus caballus* ) have established large populations in west‐central British Columbia (BC), Canada, where they overlap with native ungulates, including a declining woodland caribou ( *Rangifer tarandus caribou* ) herd. In addition, feral horses co‐occur with large carnivore species including wolf ( *Canis lupus* ) and cougar ( *Puma concolor* ). Feral horses may act as a resource subsidy for predators, potentially altering predator–prey dynamics, yet empirical observations of predator interactions with feral horses are scarce in Canada. Between 2019 and 2025, we documented 21 instances of wolf predation or scavenging of feral horses, including one direct observation of wolves actively hunting feral horses. We also documented 58 instances of confirmed feral horse predation by GPS‐collared cougars. To the best of our knowledge, these are the first published observations of wolves hunting feral horses, and the first records of cougar predation of feral horses in British Columbia. Our findings suggest that feral horses may increase food availability for these two large carnivore species, potentially facilitating elevated predation pressure on native ungulate populations via apparent competition. These novel interactions underscore the complex and far‐reaching ecological consequences of feral species. Further, they highlight the importance of incorporating non‐native prey subsidies into predator–prey management frameworks.

## Graphical abstract

In west‐central British Columbia, large populations of feral horses overlap with native species like caribou and predators such as wolves and cougars, potentially disrupting predator–prey dynamics. Between 2019 and 2025, researchers documented multiple instances of wolf and cougar predation on feral horses—the first such confirmed cases in the region. These findings suggest that feral horses may act as a food source for predators, possibly increasing pressure on native ungulates and emphasizing the need to consider non‐native prey in ecosystem management.

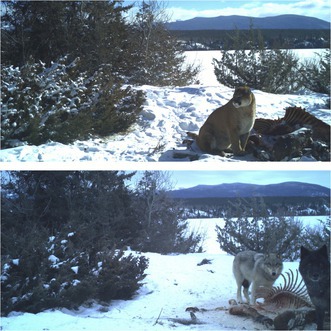

## 1 Introduction

Invasive and non‐native species are recognized as major drivers of biodiversity loss and ecosystem disruption worldwide (Díaz et al. 2019). The establishment of an abundant non‐native prey species can act as a resource subsidy for native predators, influencing their population growth rates and foraging behavior (Oro et al. 2013; Pintor and Byers 2015; Osorio et al. 2020; Ripple and Beschta 2006; DeCesare et al. 2010). These subsidies can lead to apparent competition, where subsidized predators increase predation on native species, potentially affecting conservation outcomes (Holt 1977; Courchamp et al. 1999). Feral horses ( *Equus ferus caballus* ) are a formerly domesticated species that now persist in the wild throughout much of the world (Boyce and McLoughlin 2021). Feral horses may provide an important food resource for large carnivore species such as wolves ( *Canis lupus* ) and cougars ( *Puma concolor* ), with overlap between these species confirmed in many regions. For wolves, notable areas of overlap include northern Spain (Llaneza et al. 1996; López‐Bao et al. 2013; Newsome et al. 2016) and parts of British Columbia (White et al. 2020; Parr and McCrory 2022) and Alberta (Webb 2009; Alberta Environment and Protected Areas 2023). Overlap with cougars occurs primarily in western Canada (Knopff et al. 2010; White et al. 2020), western USA (Andreasen et al. 2021), and parts of South America (Bostal et al. 2025).

In northwestern Spain, wolf predation on free‐ranging Galician Feral Ponies (*Equus ferus atlanticus*) is well documented, with these ponies representing an important prey species for wolves (López‐Bao et al. 2013; Lagos and Bárcena 2018). Conversely, in North America, documented cases of wolf predation on horses are rare and most often associated with livestock depredation. Such incidents are reported infrequently in annual publication summaries by state wildlife agencies in the United States (i.e., Idaho Department of Fish and Game 2023). Despite spatial overlap between gray wolves and domestic horses across western North America, wolf‐related livestock conflicts overwhelmingly involve sheep and cattle, with horse depredation comprising a small proportion of reported cases (DeCesare et al. 2018). Published records of wolf predation on feral horses are even less common. Available evidence is limited to a small number of reports from west‐central Alberta (Webb 2009) and the detection of horse remains in wolf scat in British Columbia (Parr and McCrory 2022). Direct observations of wolves hunting horses in the wild are exceptionally rare. To our knowledge, only a single published account exists, involving wolves hunting unattended pack horses in Jasper National Park, Alberta (Carbyn 1974; Mech et al. 2015). In remote wilderness areas, where direct observations of wolf‐horse interactions are limited, this predator–prey relationship remains poorly characterized. In such areas, feral horses may provide a resource subsidy for wolves through both predation and scavenging, potentially influencing broader predator–prey dynamics.

In contrast to wolves, records of cougar predation on feral horses are increasingly being reported, particularly in western United States (Andreasen et al. 2021; Iacono et al. 2024), western Canada (Knopff et al. 2010), and South America (Bostal et al. 2025). In western Utah, where cougars coexist with feral horses and native prey, feral horses comprised 32% of the diet of radio‐collared cougars (Iacono et al. 2024). In this study, 79% of collared cougars (23 of 29) preyed on horses across all age and sex classes (Iacono et al. 2024). Similarly, Andreasen et al. (2021) reported that feral horses accounted for 60% of cougar prey items in the Great Basin of western USA. This was higher than mule deer (a primary prey species for cougars; Robinson et al. 2002), which comprised only 29% of cougar diet, although mule deer occured at low densities in their study area. Of 13 radio‐collared cougars, 8 individuals exhibited specialization (Knopff and Boyce 2007) on feral horses, and 10 cougars routinely preyed on horses (Andreasen et al. 2021). Furthermore, in Argentina, cougar (i.e., puma) predation was determined to be an important limiting factor for feral horse populations, with foals preferentially selected by cougars (Bostal et al. 2025). Similarly, in Nevada, USA, cougar predation on feral horse foals was found to impact feral horse recruitment and population growth (Turner Jr. et al. 1992).

Together, these findings illustrate contrasting predator–prey dynamics involving feral horses and apex predators in North America. While wolves appear to prey on horses infrequently, cougars show a growing reliance on feral horses in areas where they may provide a novel, abundant food subsidy. Here, we document records of wolf predation and scavenging of feral horses on the Chilcotin Plateau (hereafter, the Chilcotin) in the central interior of British Columbia (BC). In addition, we describe records of cougar predation of feral horses in this region, which are part of an ongoing research project to understand predator–prey interactions of cougar and woodland caribou (J. Thomas, in prep). These records are primarily descriptive, yet they provide a foundation for future studies of predator–feral horse interactions and their potential implications for native prey populations (Serrouya et al. 2021) and habitat use (Beever 2003; Davies et al. 2014).

## 2 Study Area

**FIGURE 1:**
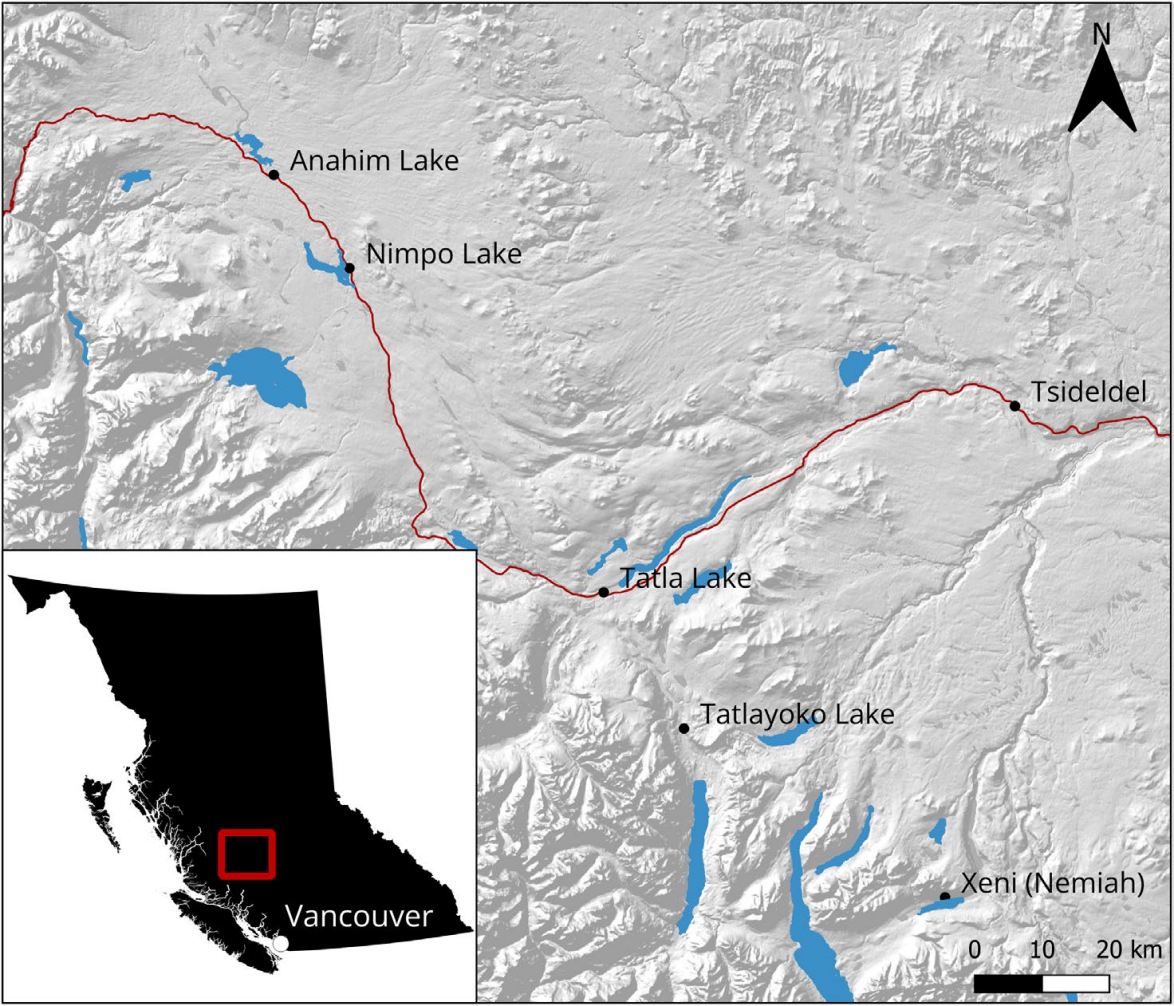
Study area on the Chilcotin Plateau, west‐central British Columbia, Canada. Highway 20 is indicated by a red line.

**FIGURE 2:**
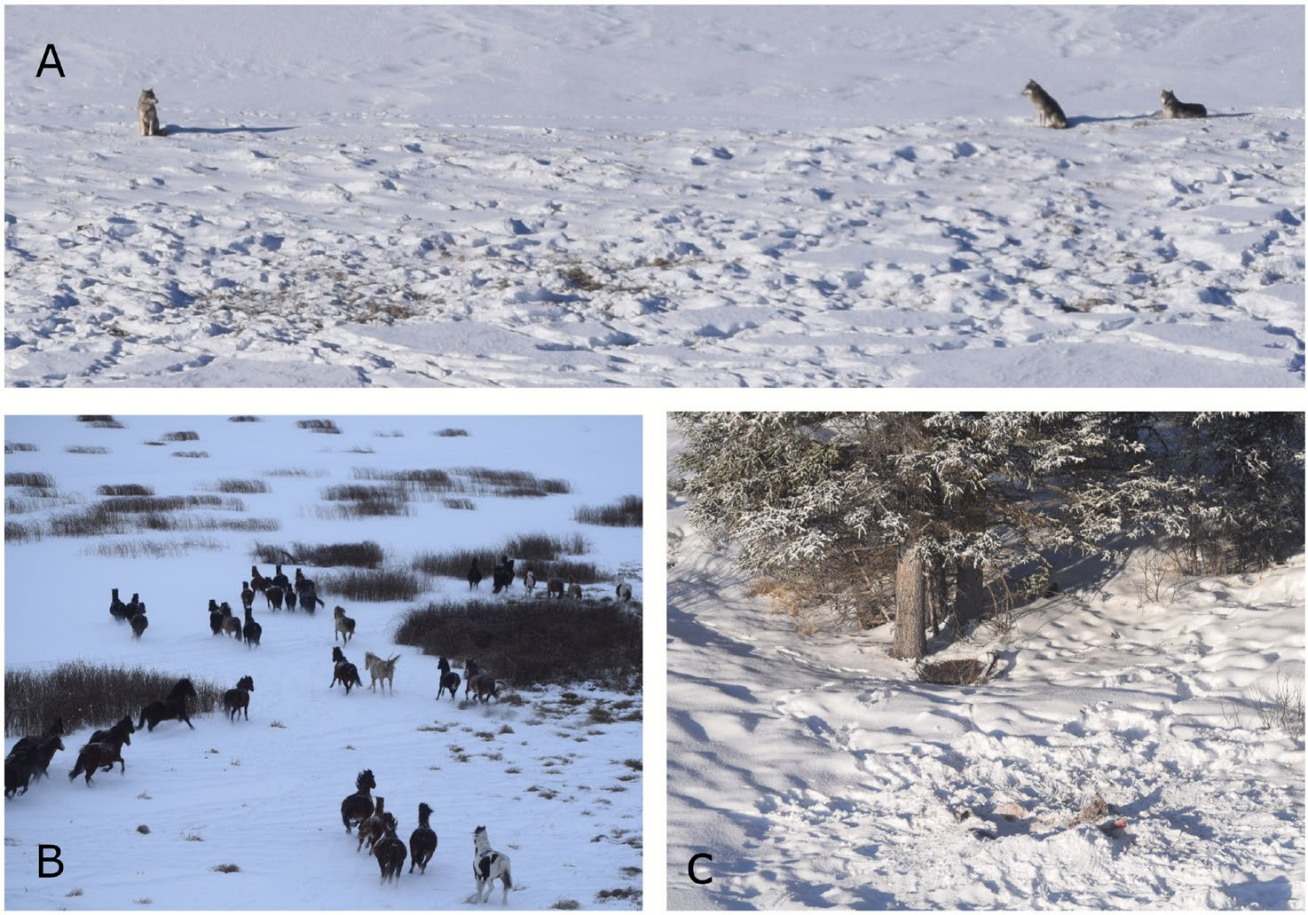
(A) Three wolves milling in a meadow approximately 200 m from a group of 14 feral horses February 10th, 2020. (B) Feral horse herd observed below helicopter during a wolf survey February 11th, 2021. (C) Feral horse carcass where a wolf was observed feeding on. A wolf bed can be seen at the base of tree, February 8th 2020. Photos by S.White.

Observations of wolf and cougar interactions with feral horses occurred in the Chilcotin region of west‐central BC, Canada—a remote area of 25,000 km^2^ (Figure 1). A large population of feral horses persist on this landscape (Figure 2b), characterized by flat to rolling terrain with elevations ranging between 800 and 2400 m. This horse population has persisted on the Chilcotin since approximately 1740 (McCrory et al. 2014) and was estimated at 2787 individuals when last surveyed in 2019 (Smith et al. 2019). Feral horses primarily occupy low elevation habitats in this system, dominated by dry forests of Lodgepole Pine ( *Pinus contorta* ) and Interior Douglas‐fir, with abundant wetland complexes and localized stands of Engelmann Spruce ( *Picea engelmannii* ). Mean daily winter temperatures of −10.5°C occur from November to February (Government of Canada 2025). Land use varies throughout this area and includes a mix of provincial parks, wilderness areas, and zones of intensive timber harvesting (Nagy‐Reis et al. 2021). Additionally, the landscape has been significantly impacted by wildfires and Mountain Pine Beetle infestations ( *Dendroctonus ponderosae* ) (Sager and Waterhouse 2015). This is a multi‐predator–prey system that includes Moose ( *Alces americanus* ), Mule Deer ( *Odocoileus hemionus* ), Mountain Goat ( *Oreamnos americanus* ), White‐tailed Deer ( *Odocoileus virginianus* ), Wolf ( *Canis lupus* ), Grizzly Bear ( *Ursus arctos* ), Black Bear ( *Ursus americanus* ), Wolverine ( *Gulo gulo* ), and Cougar ( *Puma concolor* ). Of note, feral horses in this system also overlap habitat occupied by the declining Itcha‐Ilgachuz caribou ( *Rangifer tarandus caribou* ) population (COSEWIC 2014).

## 3 Methods

To better understand predator–prey dynamics in the Chilcotin, wolf and cougar populations have been closely monitored across the Itcha‐Ilgachuz caribou range since 2019. We opportunistically documented wolf‐horse interactions through annual aerial wolf surveys (2019–2025) in support of caribou recovery, while cougar‐horse interactions were documented through a cougar GPS‐collaring study (2022–2025). We followed a census method developed by Serrouya et al. (2015) for wolf surveys across areas of low topographic relief. The method involved systematic searching for wolf tracks across a 25,000km^2^ area, following tracks until packs were detected.

From 2022 to 2025, we deployed 21 GPS collars (GmBH Vertex Lite Iridium, Vectronic Aerospace) on 17 cougars—10 males (59%) and 7 females (41%)—as part of a collaborative study investigating cougar prey selection in low‐elevation caribou winter range (J. Thomas, in prep). Cougar capture and radio‐collaring followed established provincial standards in British Columbia, following Resources Inventory Standards Committee (RISC) guidelines for cougar inventory methods and live animal capture and handling (RISC 1998a, 1998b). GPS collars were programmed to record a location every 2 h, enabling identification of potential kill sites through GPS clustering (Bacon et al. 2011; Severud et al. 2015). Similar to methods for other cougar research projects, we defined GPS clusters as ≥ 5 locations within a 100‐m radius and within a 3‐day period (Elbroch et al. 2017; Allen et al. 2023). Ground teams investigated cougar GPS clusters to identify prey species and age class. All staff involved in aerial wolf and cougar kill site investigations were experienced in determining cause‐specific mortality and distinguishing predation and scavenging events (Cristescu et al. 2022). We used evidence such as drag marks, evidence of a chase, puncture wound location and pre‐mortem hemorrhaging, sheared hair, predator sign (tracks, scat, and bed sites), and diagnostic patterns of prey consumption to identify cougar and wolf predation events. If we found insufficient evidence to be certain of predation, we classified the event as ‘probable predation’. We identified scavenging events based on evidence of other mortality sources (e.g., drowning, disease, human‐related causes, or a different predator species) or, in the case of cougars, a mismatch between the stage of carcass decomposition and the date of the GPS cluster.

We placed motion‐triggered wildlife cameras (Reconyx HP2X and Browing Patriot) at recent cougar kills (i.e., those where cougars were actively feeding or > 50% of the carcass remained) to record wildlife activity for up to 3 months post‐kill. We programmed cameras to take 3 photos during a motion‐detection event, followed by a 30 s quiet period. We used wildlife cameras to monitor cougar kitten survival and to document scavenging activity by other large carnivores such as wolves (J. Thomas, in prep).

## 4 Results

Between 2019 and 2025, wildlife cameras documented nine instances of wolves scavenging horses killed by GPS‐collared cougars (Figure 4), and two cases where both species were present at carcasses not killed by either (Table 1). In addition to wildlife camera detections, wildlife staff recorded ten instances of wolves at feral horse carcasses during winter aerial surveys conducted via helicopter and fixed‐wing aircraft, primarily in remote wilderness areas. Ground investigations confirmed wolf predation of feral horses in three cases, indicated by evidence of a chase, blood spray in the snow, and ground disturbance (Cristescu et al. 2022). In five cases, predation could not be confirmed due to carcass condition or snow obscuring evidence (Figure 2c). During wolf surveys, we also recorded six instances of wolves at moose carcasses and one observation of wolves on a mule deer carcass. Wolf group sizes ranged from one to twelve, and they were primarily observed feeding on horse carcasses on frozen lakes or in meadow systems. Two additional events in February 2020 were deemed probable wolf predation, based on aerial tracking. A pilot and wildlife crew followed wolf tracks that transitioned from walking to running, merged with horse tracks, and led to two mostly consumed horse carcasses that were 40‐m apart. Snow conditions prevented definitive conclusions. On February 12th, 2020, a pack of twelve wolves was observed actively hunting six feral horses, believed to be the first documented instance of such behavior observed in the wild. The interaction was monitored by fixed‐wing aircraft from a distance to minimize disturbance. The hunt was observed for approximately 30 min before it was interrupted by the arrival of a capture helicopter, which caused both species to disperse.

**TABLE 1:** Summary of cougar and wolf predator–prey interactions with feral horses in the Chilcotin region of central British Columbia, Canada, between 2022 and 2025.

Category	Cougar	Wolf
Predation	58 confirmed kills	3 confirmed kills
Probable predation	—	7 probable kills
Scavenging	3 scavenging events	11 scavenging events
Total observations	61 records	21 records

**FIGURE 3:**
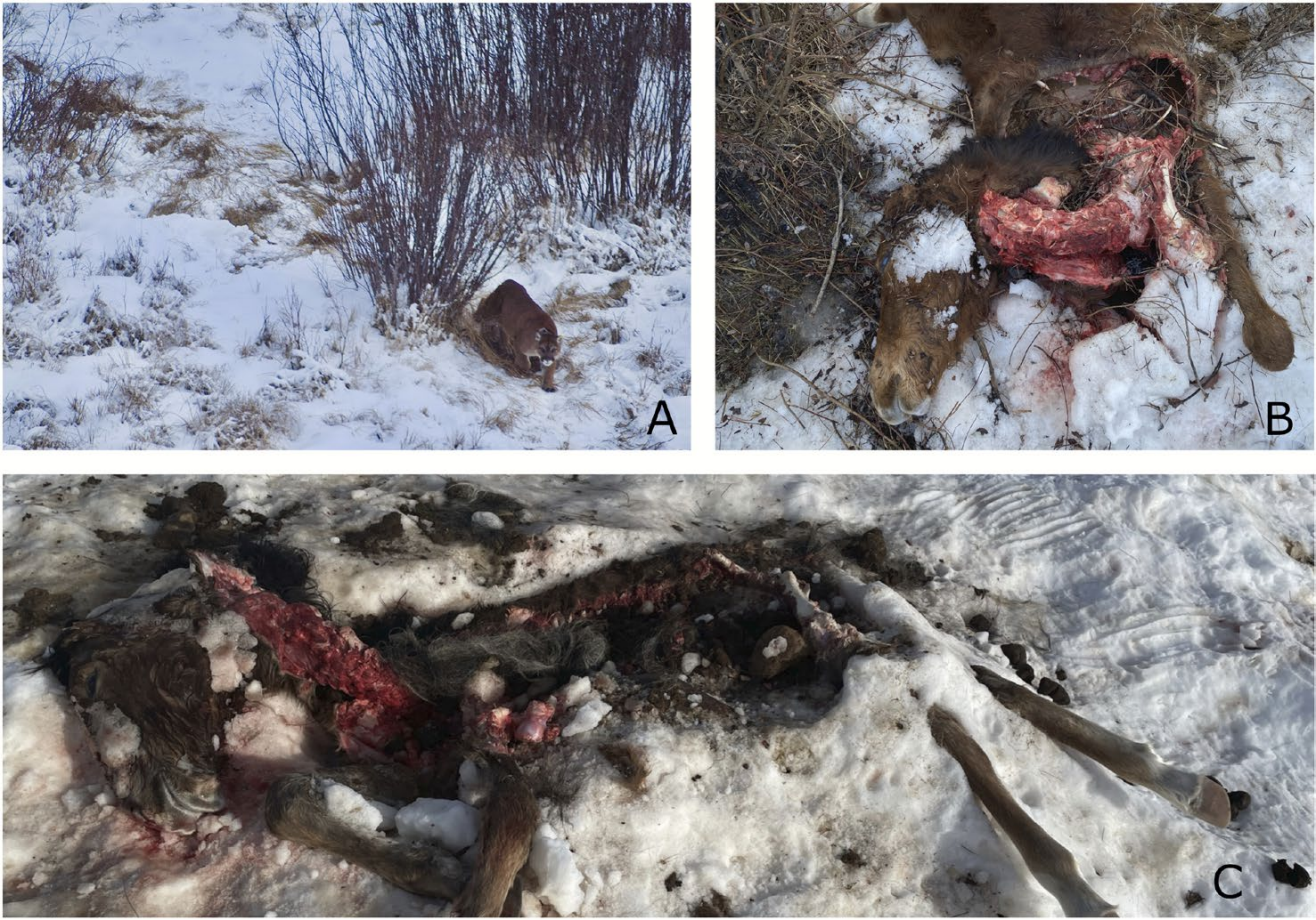
(A) Male collared cougar M3 observed from air close to a cached feral horse carcass. (B) Remains of a young feral horse carcass, killed by male cougar M1, February 2022. (C) A feral horse carcass killed and consumed by male cougar M1 February 2022. Photos S.White.

While wolf–feral horse interactions were documented opportunistically, we recorded cougar–horse interactions by investigating kill sites of GPS‐collared cougars. Since 2022, we documented 58 confirmed cougar predation events on feral horses in the Chilcotin region (Figure 3b,c). Foals comprised 60% of feral horse kills, followed by adults (21%) and juveniles (10%). Age was undetermined in 9% of cases. Eight of 17 collared cougars (47%) consumed feral horses. We recorded three additional cases of cougars scavenging on feral horse carcasses. All scavenging events involved adult horses, including one instance where a horse fell through lake ice and was scavenged by two collared cougars. We investigated a total of 383 cougar GPS‐clusters and identified prey carcasses (i.e., predation or scavenging events) at 305 of these sites. Feral horses comprised 20% of all cougar predation and scavenging events, underscoring their significance as a prey species in this ecosystem. Most horse kills were attributed to male cougars (79% of kills; Figure 3a), with one individual male responsible for 43% of confirmed kills. Feral horses accounted for 27% of prey items consumed by male cougars and 11% of prey consumed by female cougars.

**FIGURE 4:**
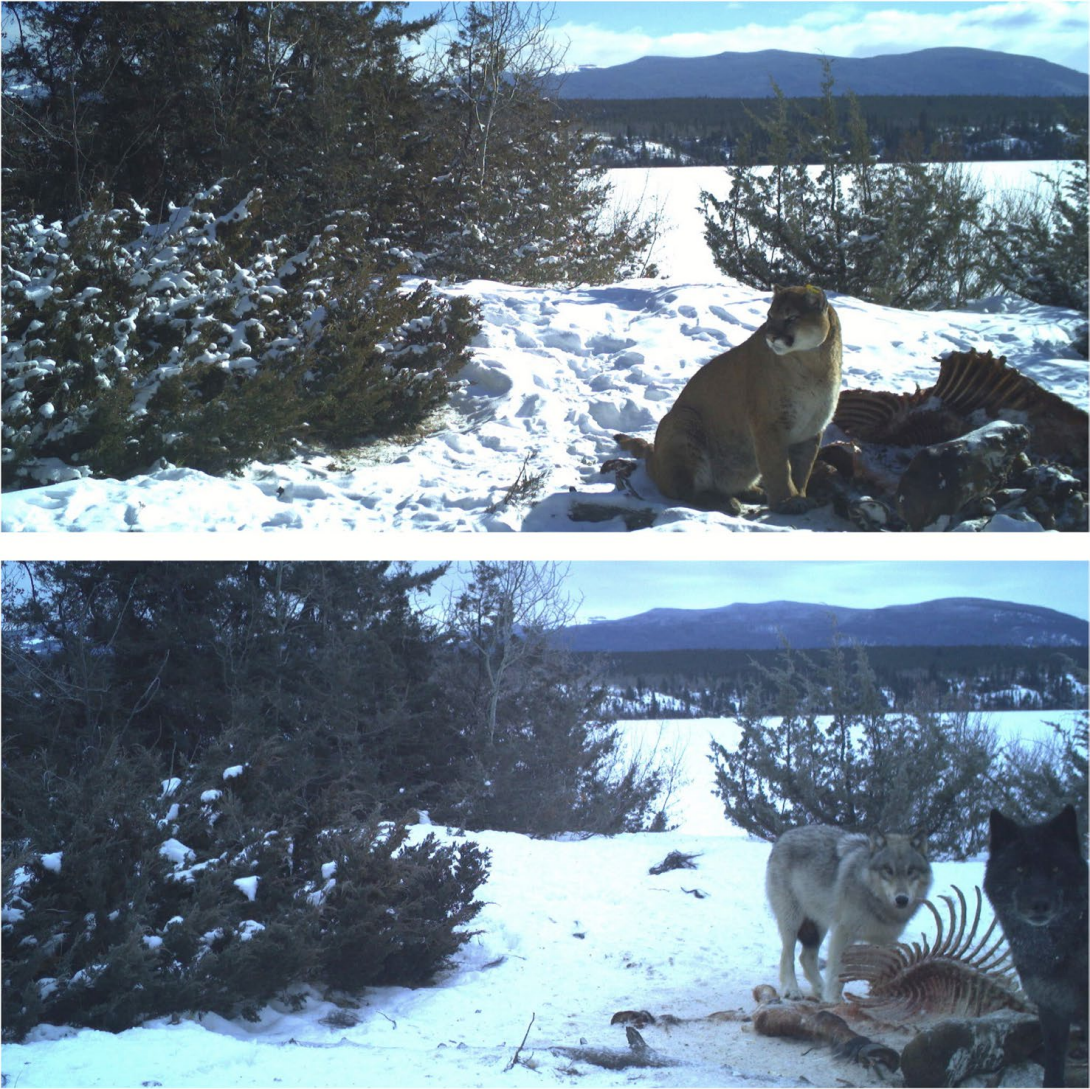
Male collared cougar M3 captured on wildlife camera at feral horse kill site February 23rd, 2023, with two wolves scavenging the same carcass March 1st, 2023. Photos: J. Thomas.

## 5 Discussion

Feral horses in BC are not recognized as wildlife and are not managed under the Wildlife Act. Although they are classified as livestock under the Livestock Act (British Columbia 1982), they remain functionally unmanaged, and population monitoring is not required. This has resulted in a significant knowledge gap regarding their relationship with native species and habitat in BC. This differs significantly from western USA, where feral horse populations are managed under the Wild Free‐Roaming Horses and Burros Act of 1971 (United States 1971), prompting more research into their ecology and impacts on native ecosystems.

Our records of wolves and cougars preying or scavenging on feral horses may suggest that abundant feral horses provide a resource subsidy to native predators in central BC (Newsome et al. 2015). While our data on wolf‐horse interactions are limited due to low survey effort, they raise the possibility of an important predator–prey relationship. This may be particularly true during harsh winters when feral horses are vulnerable to predators due to reduced food availability, increased energy demands (Berger 1983; Garrott and Taylor 1990; Harvey et al. 2021), and deep snow (Huggard 1993; Sullender et al. 2023). Immediately south of our study area, Parr and McCrory (2022) documented a high frequency of horse remains in 122 wolf scats collected over 5 years. However, the presence of domestic horse carcasses left on the landscape by people in their study area may have inflated their findings. Nonetheless, our observations further support their conclusion that horses provide an important food source for wolves in this system. To better understand wolf–feral horse dynamics, future analyses of wolf habitat and prey selection relative to feral horse distribution could clarify the extent of this relationship (Hebblewhite and Merrill 2008; Kittle et al. 2015). Planned stable isotope analysis on both cougar and wolf samples collected since 2019 may provide further insight into how prevalent consumption of feral horses is in this system for both these predators. Furthermore, cougar population information derived from DNA genotyping may provide complementary insight into predator population dynamics in this system.

Our observations suggest that feral horses, particularly foals (i.e., < 1 year old), are an important prey item for cougars in our study area. This aligns with findings that cougar predation of foals can be a limiting factor for feral horse populations (Turner Jr. et al. 1992; Bostal et al. 2025). In our study area, male cougars were primarily responsible for horse predation, contrasting with other regions where reproductive females showed near‐complete reliance on feral horses year‐round (Andreasen et al. 2021). Cougars preyed on adult horses in addition to younger age classes, reflecting the ability of male cougars to take down prey substantially larger than themselves (Clark et al. 2014).

Feral horse predation may have broader ecological consequences for native prey species, through the mechanism of apparent competition. Apparent competition could occur if native predator populations (e.g., wolves and cougars) are supported by abundant feral horses, with negative consequences for native prey such as moose and woodland caribou (Wittmer et al. 2013; Tjaden‐McClement et al. 2025). This hypothesis is the focus of ongoing research in central BC. As noted by Boyce and McLoughlin (2021), feral horses may pose an elevated risk as apparent competitors because their populations can be decoupled from natural regulatory processes. High adult survival and reproductive rates may inflate feral horse abundance under some management regimes, thereby subsidizing predator populations beyond levels supported by native prey alone. In addition, feral horses have volatile population dynamics, which could increase predation pressure on sympatric native ungulates during horse population lows (Boyce and McLoughlin 2021).

Further research is needed to fill critical knowledge gaps about predator‐feral horse interactions. This is especially important in areas where feral horses overlap with declining or threatened native species, such as the Itcha‐Ilgachuz caribou population in the Chilcotin. Beyond their role as a food source, feral horses have been shown to cause significant habitat degradation in arid and semi‐arid environments, such as those present in central BC. These impacts include reduced plant cover, loss of native vegetation, decreased small mammal diversity, soil erosion, and riparian damage (Beever and Brussard 2004; Ostermann‐Kelm et al. 2009; Davies et al. 2014). Investigating these impacts could inform future management decisions and contribute to a broader understanding of feral horse effects across ecosystems. Given their ecological significance and potential impacts on native species, current classifications under the Livestock Act may not fully reflect their role or the management challenges they present. Greater knowledge of feral horse population dynamics and interactions with predators and native ungulates could guide future research and management strategies, supporting more effective conservation planning.

## Author Contributions

**Shane C. White:** conceptualization (lead), funding acquisition (lead), investigation (lead), project administration (equal), writing – original draft (lead). **Julie Thomas:** investigation (supporting), methodology (supporting), project administration (equal), writing – review and editing (supporting). **Caroyln Shores:** conceptualization (supporting), funding acquisition (supporting), project administration (supporting), writing – review and editing (supporting). **Kathi Zimmerman:** funding acquisition (supporting), project administration (supporting), supervision (lead), writing – review and editing (supporting).

## Conflicts of Interest

The authors declare no conflicts of interest.

## Data Availability

Data sharing not applicable to this article as no datasets were generated or analysed during the current study.
