# Correction to “Where Dinner Roams: The Role of Feral Horses as a Resource Subsidy for Wolves and Cougars in West‐Central British Columbia”

**DOI:** 10.1002/ece3.73269

**Published:** 2026-03-17

White, S. C.,J. Thomas, C. Shores, and K. Zimmerman. 2026. “Where Dinner Roams: The Role of Feral Horses as a Resource Subsidy for Wolves and Cougars in West‐Central British Columbia.” *Ecology and Evolution* 16, no. 2: e73089. https://doi.org/10.1002/ece3.73089.

The name of the third author in the author list, “Caroyln Shores”, was misspelled. This should have read “Carolyn Shores”.

This has been corrected in the published article.

We apologize for this error.

